# Predicting the distribution of COVID-19 through CGAN—Taking Macau as an example

**DOI:** 10.3389/fdata.2023.1008292

**Published:** 2023-01-25

**Authors:** Liang Zheng, Yile Chen, Shan Jiang, Junxin Song, Jianyi Zheng

**Affiliations:** Faculty of Humanities and Arts, Macau University of Science and Technology, Macau, Macao SAR, China

**Keywords:** urban public health, machine learning, epidemic hotspots, CGAN, risk assessment

## Abstract

Machine learning (ML) is an innovative method that is widely used in data prediction. Predicting the COVID-19 distribution using ML is essential for urban security risk assessment and governance. This study uses conditional generative adversarial network (CGAN) to construct a method to predict the COVID-19 hotspot distribution through urban texture and business formats and establishes a relationship between urban elements and COVID-19 so that machines can automatically predict the epidemic hotspots in cities. Taking Macau as an example, this method is used to determine the correlation between the urban texture and business hotspots of Macau and the new epidemic hotspot clusters. Different types of samples afforded different epidemic prediction accuracies. The results show the following: (1) CGAN can accurately predict the distribution area of COVID-19, and the accuracy can exceed 70%. (2) The results of predicting the COVID-19 distribution through urban texture and POI data of hospitals and stations are the best, with an accuracy of more than 60% in experiments in different regions of Macau. (3) The proposed method can also predict other areas in the city that may be at risk of COVID-19 and help urban epidemic prevention and control.

## 1. Introduction

Since the outbreak of COVID-19 at the end of 2019, it has become a continuous global epidemic. It is one of the deadliest epidemics in human history and is also a closely watched urban public health governance issue. As of July 29, 2022, more than 574 million confirmed cases have been globally reported, and more than 6.395 million people have died, with the number continuously increasing. During the peak of the pandemic, the work order of hospitals and doctors as well as the care of patients without COVID-19 became extremely challenging, placing considerable strain on the city's public health administration (Chibbaro, 2020; Ganau, 2020). Owing to the development of information technology and urban big data, epidemic prediction models can be constructed data using the spatiotemporal information and future epidemic situations can be simulated at fine spatial scales.

As early as May 25, 2020, a team from Lanzhou University in China built an epidemic prediction system using the SIR prediction model, which is also the world's first epidemic prediction system. Subsequently, based on the development situation of COVID-19 in various places and the characteristics of cities, various scholars have established traditional dynamic and statistical models to simulate and predict the COVID-19 spread and distribution. Among them, the infectious disease dynamics research on COVID-19 has been mainly developed based on the classic compartmental models, which is also the mainstream prediction and simulation method (Dogan et al., 2021; Pei et al., 2021). Compartmental models can be classified into several types, such as SIR (Bjørnstad et al., 2002), SIER (Qian et al., 2020), SEIRS (Wu et al., 2020), and SEIHR (Sanyi et al., 2020). They mainly analyze crowd flow and spatiotemporal information and provide an interaction model between individuals and complex environments (Chen-Charpentier and Stanescu, 2010; Alsayed et al., 2020; Yu et al., 2020). However, they excessively rely on the refinement degree of the data, the modeling is extremely complicated, the parameters dynamically change at different stages of the epidemic, and the simulation accuracy of the spatial distribution of confirmed cases is low. Statistical models mainly use historical data to predict and simulate future epidemics, with high accuracy and simple data processing, such as the logistic model based on spatiotemporal big data (Song et al., 2020), the generalized spatiotemporal autoregressive model (Pasaribu et al., 2021), and the ordinary differential equations for population zoning models (Miranda et al., 2021). Moreover, researchers have developed mathematical models of infectious disease dynamics to aid in the identification of potential intervention strategies (Balak et al., 2021).

With the development of artificial intelligence technology, the value of machine learning (ML) algorithms has been demonstrated. Different algorithms are trained through ML and prediction models based on real-time data are established, which have been widely used to predict the inflection point and end time of the epidemic. Existing studies have shown that ML can well simulate the spatial and temporal changes and accurately predict the evolution of high-fidelity numerical simulations. Compared to the traditional dynamic models whose prediction results deviate due to the different parameters and modeling data in each stage of the epidemic, ML can continuously update simulations based on real-time data (Hao et al., 2020; Pasaribu et al., 2021; Silva et al., 2021). Simultaneously, the multi-disciplinary cross model of ML combined with the geographic information system model can well conduct spatial evolution simulation research. For example, the time-domain difference model (Cong et al., 2020), quintuple model (Shen et al., 2020a), and random forest model (Ong et al., 2018) afford small prediction errors, showing the feasibility of ML methods in spatial simulation.

Although the above ML methods solve the limitations of some traditional epidemic prediction models, the structure of ML models is relatively complex during modeling, they can only output a single variable, and they do not consider the impact of crowd behavior and control efforts on the spatial distribution of the epidemic. Therefore, considering multiple factors such as the distribution of business types, footprints, and hotspots in the city, further data mining through ML is required to obtain accurate prediction methods for the current development of COVID-19 in cities.

## 2. Research methodology and data sources

Based on the image synthesis technology in ML and computer vision (CV), this study predicts the footprint heat map of COVID-19 using urban morphology maps and POI heat maps. First, the urban morphology maps and POI heat maps of the Macau Peninsula^1^ are used as training set A, and the corresponding COVID-19 footprint heat maps are used as training set B. Conditional generative adversarial networks (CGANs) are used (Mirza and Osindero, 2014) for training; subsequently, in the image translation using training sets A and B, the generator and discriminator are confronted, thereby improving the accuracy of image translation (Chrysos et al., 2018). Finally, the ability of the urban morphology maps and POI heat maps to predict the urban COVID-19 footprint hotspots is studied.

### 2.1. Sample processing

**Figure 1:**
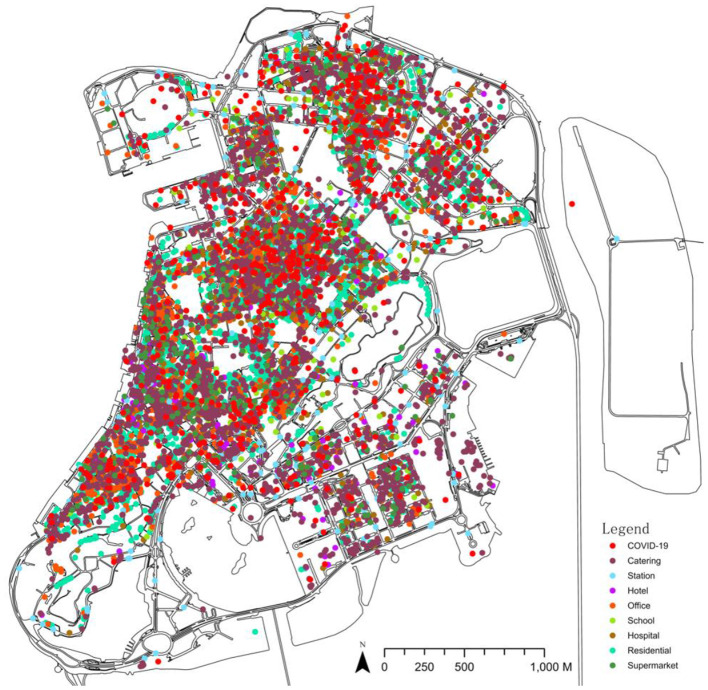
The footprint and POI distribution of the COVID-19 epidemic on the Macau Peninsula.

As the test dataset for training the network, the Macau map data of the Macau Cartography and Cadastre Bureau as well as the Macau POI data of the AutoNavi map are selected (Table 1). The footprint hotspot data of COVID-19 are generated based on the statistics of the footprint report of a total of 500 patients in Macau (from mid-June to early July 2022), which was fully disclosed by the Macau Health Bureau. The report investigated the location and scope of each COVID-19 patient's pre-illness activities. Footprints from 3,982 specific addresses are eliminated (including 3,265 in the Macau Peninsula), and the addresses are converted into latitude and longitude coordinates using Google Maps API Web Services. They are then input into ArcGIS Pro to generate hotspot data. CGAN requires paired datasets for training; thus, to ensure that the data have one-to-one correspondence, the above data are uniformly corrected into the Observatorio Meteorologico 1965 Macau Grid (Figure 1).

**Table 1:** Classification of map POI data.

**POI category**	**Number of points (pieces)**	**POI category**	**Number of points (pieces)**
Catering	3,443	Supermarket	443
Hotel	145	Office	691
Station	268	School	148
Hospital	1,819	Residential	2,638

**Figure 2:**
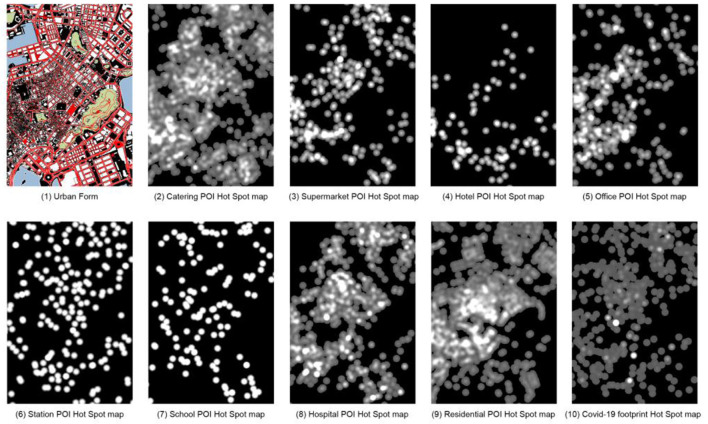
Area of sample selection.

Since ML methods require a large number of samples to obtain accurate experimental data, the sample images are divided into grids with a size of 512 × 512 pixels, and each image slice has an area of about 4 ha. In the first test, since the Macau Peninsula is surrounded by sea on three sides, using the entire Macau Peninsula as the scope, slicing according to the network results in a large number of slices with no useful information in the edge area, which considerably impacts the model accuracy. It has been verified by trial and error that using the information-rich region in the central part of the Macau peninsula is beneficial for training CGAN; thus, the central part of the Macau peninsula is selected. In the processing of the epidemic trajectory map, using the above statistical coordinate points, a search radius (search area) of 10 m is set to generate a hotspot map wherein different colors represent the density of the distribution of COVID-19 patients, with lighter colors representing higher densities, darker colors representing lower densities, and black color representing no distribution (Figure 2).

In the processing of the Macau map, for data simplification, various elements in the map are represented in different colors and presented in the form of colored pictures (Shen et al., 2020b). In this study, five colors are used to represent the map elements: roads are red (*R* = 255, *G* = 0, *B* = 0), green spaces are green (*R* = 0, *G* = 255, *B* = 0), water is blue (*R* = 0, *G* = 0, *B* = 255), buildings are white (*R* = 255, *G* = 255, *B* = 255), and land is black (*R* = 0, *G* = 0, *B* = 255). These five colors represent most of the content in the city map.

In the selection of POI samples, eight types of representative POIs are selected based on the previous studies on the importance of POIs (Cao et al., 2018) and the characteristics of Macau cities, namely restaurants, supermarkets, hotels, offices, stations, schools, hospitals, and residences. Since the COVID-19 virus can be transmitted by aerosols in an environment of 10 m (Setti et al., 2020), the heat map is drawn with a radius of 10 m. As shown in Figure 2, significant differences exist in the distribution of hotspots in various categories: ① It is relatively uniform in restaurants, hotels, residences, and epidemics. ② It is moderate in supermarkets and offices. ③ It is concentrated and sparse in stations and schools. ④ It is minimal in hotels.

**Figure 3:**
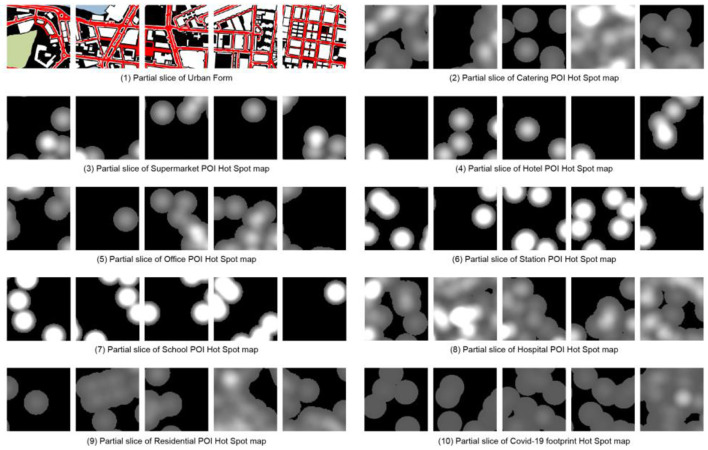
Sample Sectioning.

As is well known, the quality of image datasets is critical to the success of image generation. Therefore, the 10 types of pictures are divided according to the size of 512 × 512 pixels. After weighing the quality and quantity, a 9 × 6 grid is obtained. Each type of picture is cut into 54 pictures, yielding a total of 540 samples (Figure 3). Using the above samples, the COVID-19 footprint hotspot slice map is combined with pictures of other categories as ML materials to train nine different weight files. According to the CGAN characteristics, the input data are divided into training sets A and B. Training set A contains basic information about the city, including roads, green space, water, buildings, and land, and POI data that indirectly reflect the nature of urban land. Training set B contains the distribution of COVID-19 hotspots as the prediction target.

### 2.2. Generative adversarial network (GAN)

**Figure 4:**
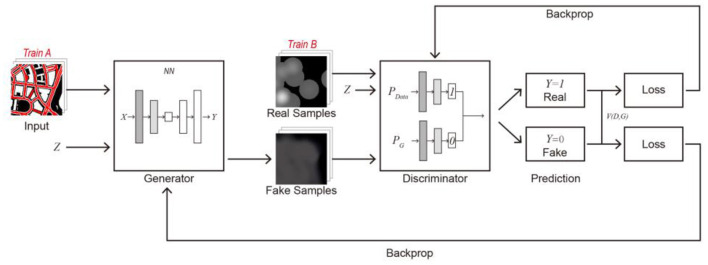
Principle of CGAN.

This study uses CGAN to predict the distribution of urban epidemic hotspots. CGAN is a variant of generative adversarial networks (GANs). Consistent with the original GAN, CGAN is mainly composed of two adversarial models: a generator responsible for generating images and a discriminator for judging the authenticity of the generated images. As shown in Figure 4, the main principles are as follows: ① The generator generates fake pictures according to the input picture (Train A) and random vector (Z). ② The discriminator determines another set of corresponding images (Train B) and random vectors as real images and simultaneously discriminates with the fake images input by the generator. The real image is denoted as 1, and the fake image is denoted as 0. ③ If the generated picture is judged to be false, the discriminator returns the deviation value between the false picture and the real picture to the generator; the generator learns so that it can generate a picture that is closer to the real picture. In contrast, if the discriminator judges that the generated image is real, the discriminator will continue to learn from the training set to improve its recognition ability. ④ Through adversarial training, the generator can finally generate fake and real pictures to achieve the prediction target.

### 2.3. Image comparison method

Reliable methods for quantitatively and qualitatively assessing and comparing the quality of the generated results of GANs are still lacking (Borji, 2019). For example, the diversity of practical needs and the lack of a method for comparing images with similarity that is equivalent to human perception make it difficult to evaluate generative models (Theis et al., 2015). Therefore, based on the characteristics of the training set and the generated result images, this study adopts two methods of image similarity comparison: radial variance hash and histogram comparison. Utilizing additional image comparison techniques not only enables more precise quantitative evaluation of the quality of the generated results, but also facilitates the identification of patterns and correlations between samples.

Radial variance hashing judges whether two images are similar by taking the global hash of the images and comparing the Hamming distance of the hashes of two images. The more similar the two images are, the smaller is the Hamming distance of the hash values (Shuo-zhong and Xin-peng, 2007). The principle of the hash value calculation algorithm is to perform lossy compression on the original data. The fixed word length after lossy compression can be used as a unique identifier for identifying the original data. This unique identifier is the hash value. Different image hashing algorithms exist according to the different ways of calculating the image hash value. Herein, the radial variance hash value algorithm is mainly used to compare the similarity between the sample images of the training set to explore the relationship between the ML results and the correlation of similarities among the sample images.

Histogram comparison measures the similarity between the histograms of two images according to the standard distance metrics. The image histogram contains rich image detail information, which reflects the probability of the distribution of the image pixels. The number of pixels in the intensity value of each pixel is counted, and the correlation between the two image histograms is calculated using the correlation calculation formula (Srinivas et al., 2020). Unlike the radial variance hash value, the histogram can well reflect the probability distribution of the gray value of the image and has a higher similarity for images with similar brightness and darkness. Herein, the COVID-19 trajectory prediction results are presented as grayscale heat maps. The comparison of histograms, which are highly sensitive to the grayscale values of images, well reflects the accuracy of the model predictions.

### 2.4. Model training

The experiment comprises 10 different kinds of training sets, which are divided into training sets A and B. Training sets A and B are the feature and target images, respectively. Two training ensembles are used to train the ML model, with a total of nine sets of models. The model is trained for a total of 200 epochs, the gradient is evaluated using the Adam optimizer, and the update step size is calculated. Due to the limited number of training sets, the batch size is set as 2 and the learning rate is set as 1e-3 for the first 60 runs. Then, the backbone feature extraction network is frozen for training, thereby accelerating the convergence speed and preventing the destruction of the pre-training weight. The learning rate is set to 1e-4 for the last 140 times, and the backbone feature extraction network is unfrozen. The entire model is further trained with a smaller initial learning rate, thereby speeding up the training time of the entire network. A weight file is saved every 20 iterations. After training, the weight file with the best loss value is selected for the prediction. The above method is used to set up and train the model to find the connection between training sets A and B. In this way, training set B can be output by only inputting training set A, and the goal of predicting the trajectory distribution of the new cluster can be achieved. The results of the model training are shown in Table 2.

**Table 2:** Model training results.

**Samples**		**Loss**				
**Train A**	**Train B**	**G_GAN**	**G_GAN_Feat**	**G_VGG**	**D_real**	**D_fake**
Urban form	COVID-19 Hot Spot map	1.691	4.156	2.611	0.062	0.038
Catering POI		2	11.357	3.991	0.025	0.021
Supermarket POI		0.564	11.056	4.934	0.051	0.483
Hotel POI		1.526	8.456	3.507	0.104	0.063
Office POI		1.031	2.86	2.589	0.313	0.195
Station POI		0.873	3.545	1.92	0.231	0.301
School POI		1.568	5.397	3.289	0.104	0.052
Hospital POI		1.156	4.437	3.009	0.126	0.172
Residential POI		1.739	11.403	3.582	0.015	0.03

## 3. Results analysis and discussion

### 3.1. Similarity of heat maps

**Figure 5:**
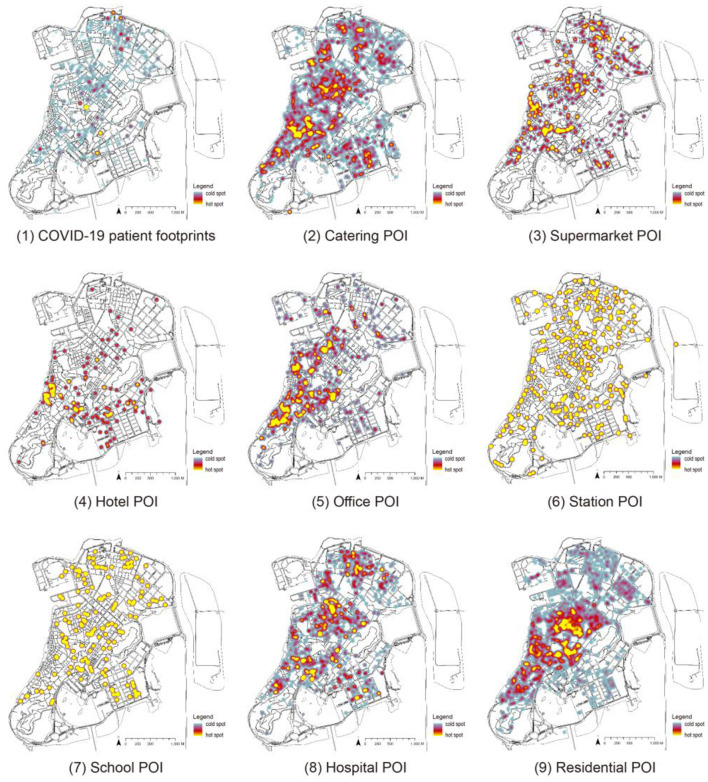
COVID-19 epidemic footprint and heat map of different types of POIs.

First, through the footprint distribution of COVID-19 infected people on the Macau Peninsula is superimposed on the residents' mobility hotspots of various major social functions in Macau, and then, the distribution map is obtained (Figure 5). The blue areas in the figure represent the cold spots of the POI heat map and the density of COVID-19 infections, and the yellow areas represent the hotspots. The overall distribution map of the footprints of infected people shows that the footprints of infected people almost cover most of the Macau Peninsula. Among them, three regions are relatively concentrated: the northern, central, and southern regions. The remaining heat maps show that the distribution areas of restaurants, supermarkets, and hospitals are relatively similar, mainly concentrated in the above three regions. The distribution areas of schools and stations are similar and basically homogeneous. The residences are mainly distributed in the central area.

After determining the footprint distribution of COVID-19 and the heat distribution map of each functional category, the following table is obtained by analyzing and comparing the epidemic footprint and the radial variance hash of various POI heat maps (Table 3). The similarity between the COVID-19 footprint and various POI heat maps is described by the compared hash values, that is, the regional overlap between the two; the pink and blue highlighted values in the table represent high and low overlaps, respectively.

**Table 3:** Comparison of COVID-19 footprint and different types of POI similarity.

	**COVID-19**	**Catering**	**Supermarket**	**Hotel**	**Office**	**Station**	**School**	**Hospital**	**Residential**
COVID-19	1	0.62	0.89	0.88	0.86	0.92	0.94	0.80	0.62
Catering	0.62	1	0.67	0.66	0.76	0.67	0.71	0.73	0.66
Supermarket	0.89	0.67	1	0.91	0.90	0.92	0.90	0.86	0.63
Hotel	0.88	0.66	0.91	1	0.90	0.93	0.88	0.81	0.55
Office	0.86	0.76	0.90	0.90	1	0.89	0.89	0.87	0.68
Station	0.92	0.67	0.92	0.93	0.89	1	0.92	0.84	0.62
School	0.94	0.71	0.90	0.88	0.89	0.92	1	0.81	0.62
Hospital	0.80	0.73	0.86	0.81	0.87	0.84	0.81	1	0.68
Residential	0.62	0.66	0.63	0.55	0.68	0.62	0.62	0.68	1

Table 3 shows that the distribution of the COVID-19 epidemic has the highest overlap with the areas of station and school categories, indicating that stations and schools are high-risk infection areas. Food and beverage categories have a low risk of infection. The high overlap between supermarket and station and school categories as well as between the hotel and station categories denotes that supermarkets and hotels have a high risk of infection spread, that is, they are potential risk infection areas. The reasons for this result are as follows:

① For high-risk areas, since people are more concentrated and the short-term flow has high frequency, the virus rapidly spreads (0.92–0.94).
② For the potential risk area, due to the high degree of activity sequence correlation with the function of the high-risk area, the risk of becoming a secondary transmission area of the epidemic is relatively high (0.9–0.93).

### 3.2. Accuracy and stability of the model

**Figure 6:**
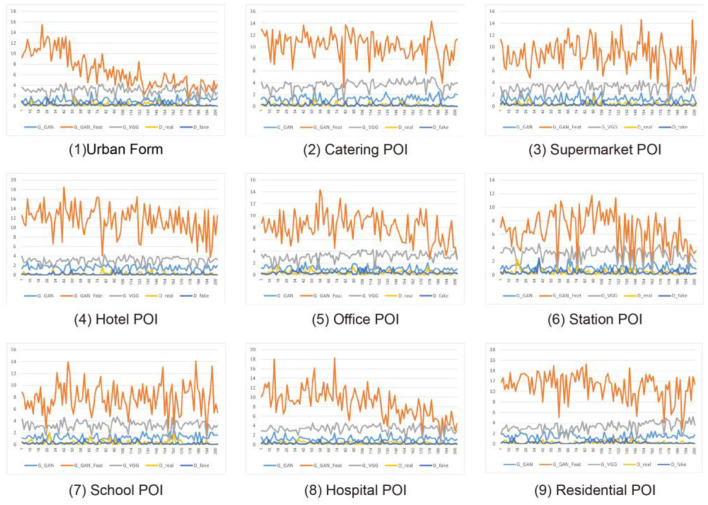
Iterative training LOSS numerical statistics.

For the accuracy and precision of model training, this study improves the accuracy and stability of recognition and learning by performing multiple iterations of convolution training on the model. However, depending on the input information, the final accuracy of the ML method differs. As shown in Figure 6, the orange line represents the fluctuation curve during the ML process, that is, the stability of the learning result. After inputting different types of city information into the ML method, the ML results based on urban form elements exhibit the smallest fluctuation range and the highest stability after 200 iterations of learning. The fluctuation range of the fluctuating curve learned based on the POI heat map of the hospital and station significantly decreases after 200 iterations of learning, proving that the learning effect is good. The learning results based on the remaining POI heat maps have different degrees of volatility, which means that the learning results are less stable.

Note that the stability of ML does not represent the accuracy of the learning results of the model. For the stability of model training, the stability of ML results varies according to the input conditions and the completeness of the input data and indirectly reflects the correlation between the learning conditions and target results. Taking the different input data in Figure 6 as an example, when the urban morphological fabric is used as the input data, the stability of the model results after 200 iterations of learning shows that urban fabric elements significantly impact the COVID-19 distribution; that is, there is a strong correlation between them. The stability of the model learning results when the POI heat maps of the hospital and station are used as input data shows that the station and hospital affect the COVID-19 distribution. The stability of the learning results of the model when the POI heat maps of the remaining categories are used as input data indicates that they are relatively weakly related to the COVID-19 distribution.

**Figure 7:**
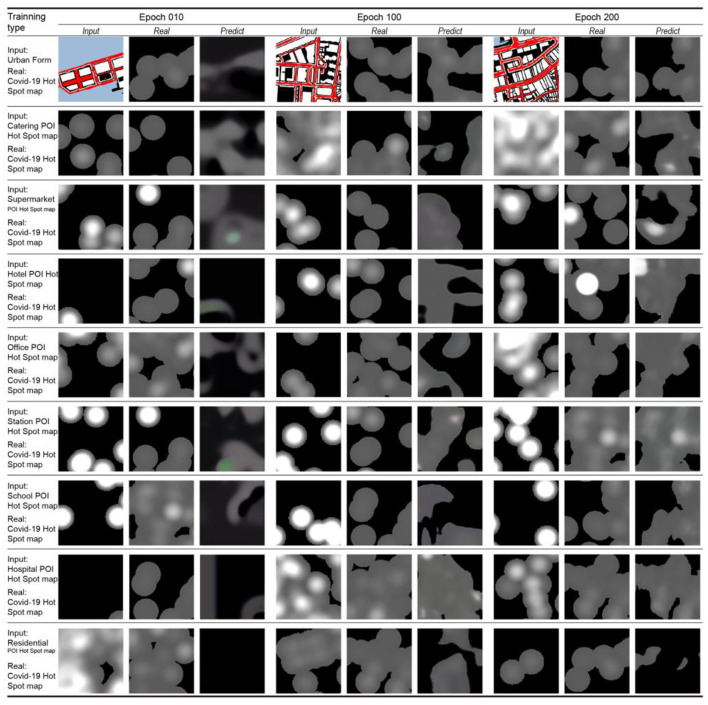
Diagram of iterative training process.

Figure 7 displays the comparison of iterative training results for model learning accuracy. Three comparison groups (columns) performed 10, 100, and 200 iterations of learning for different categories of city information (rows). Under the condition of 10 iterations of learning, the learning results are blurred and less accurate than the real COVID-19 footprint map. The results improved under the condition of 100 iterations of learning, but the accuracy is still not ideal. Under the condition of 200 iterations of learning, the similarity between the results of basic learning and the actual COVID-19 footprint is high. This shows that under the condition of maximizing the cost saving of the machine load and learning time, the improvement of the accuracy of this ML model can basically meet the target requirements after 200 iterations of learning.

### 3.3. Model application and analysis

**Figure 8:**
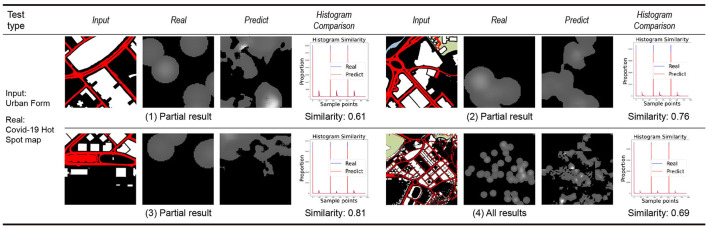
Taipa urban morphology prediction results.

**Figure 9:**
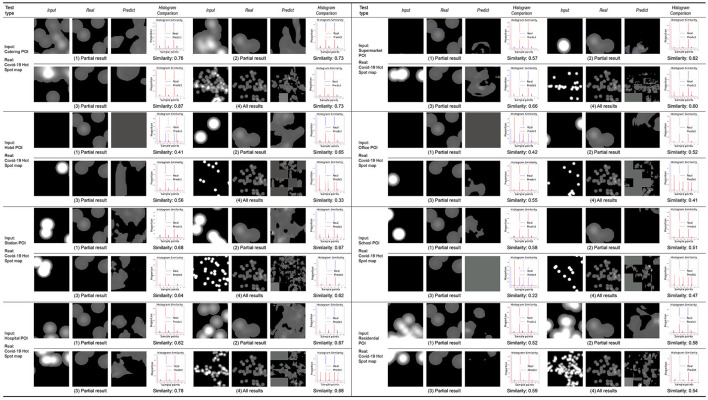
The heat map prediction results of different types of POIs in Taipa.

By training the machine to learn the city information data of the Macau Peninsula region, its connection with the distribution map of COVID-19 is established. Using the nine weighted models trained on the Macau Peninsula training set, predictions are made for the same region of the Taipa area, Macau, and the predicted distribution results of COVID-19 for the Taipa area are obtained by the machine. The prediction results are divided into two categories: prediction results obtained by inputting the texture elements of the urban form (Figure 8) and prediction results obtained by inputting the POI heat maps of different categories for learning and training (Figure 9).

The first three groups in each category display the learning results of some small-scale slices in the learning process. The fourth group displays the overall learning results of the target area after splicing the slices. “Input” in each group is the city information data of the target area. “Real” is the actual epidemic distribution of the target area, which is only used as a reference for comparing the results and does not participate in the learning and training. “Predict” is the prediction result of the COVID-19 distribution in the target area by the machine.

The prediction results obtained after learning the texture elements of the urban form have a high similarity with the actual COVID-19 distribution, reaching 70% in general. Compared to the prediction results of the 1–3 groups of small-scale slices, the prediction results include the main distribution points and regions of the COVID-19 footprint, and simultaneously, a certain degree of connection is made between different points. Compared to the actual COVID-19 distribution, the predicted results more accurately reflect the boundary of the city's block and road, and they have more practical guiding significance than the point map of the original COVID-19 distribution.

According to the prediction results obtained after inputting the POI heat maps of different categories for learning and training, the POI heat maps of different categories in the input considerably impact the learning results of the machine and the accuracy of the model prediction results. The volatility is relatively large. Based on the input of POI heat maps of offices, schools, and hotels, the accuracy of the prediction results of the COVID-19 distribution is generally lower than 50% (41% for offices, 47% for schools, and 33% for hotels). Prediction results based on the residential POI heat map input have a median accuracy of 54%. The accuracy of the prediction results based on the POI heat map input from hospitals, restaurants, supermarkets, and stations is high, all higher than 60% (68% in hospitals, 73% in restaurants, 60% in supermarkets, and 62% in stations). Analysis shows that the reasons for the difference in the above prediction results are as follows:

① Due to the limitation of the distribution density of the POI heat map at the input end, the machine will yield erroneous and invalid prediction results when making learning predictions, that is, the all-gray prediction results in “predict”.

② Due to the limitation of the amount of information in the COVID-19 distribution map, the machine cannot establish more accurate association learning through more association conditions in the process of establishing association learning.

**Figure 10:**
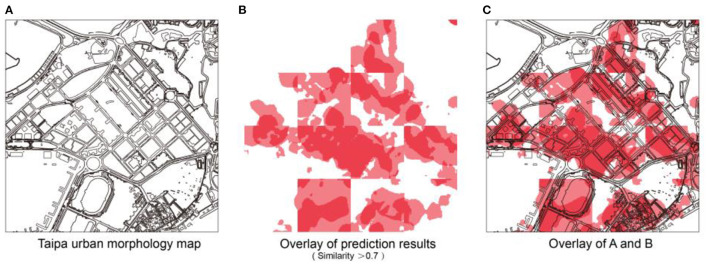
Combined forecast results: **(A)** Taipa urban morphology map; **(B)** Overlay of prediction results (Similarity > 0.7); **(C)** Overlay of A and B.

After forecasting the COVID-19 distribution in the target area of Taipa, the city base map of the area is superimposed on it to afford a schematic of the final forecast of the COVID-19 distribution in the target area (Figure 10). It has a more intuitive reference value for future urban planning management and epidemic risk control in urban units.

## 4. Conclusion and outlook

Based on ML, this study used the urban texture map and the heat map of eight types of POIs to predict the COVID-19 distribution in Macau. The following conclusions can be drawn:

(1) CGAN can accurately predict the COVID-19 distribution and can effectively predict other areas in the city that may be at risk of COVID-19. This method has applicability and reference for public health governance and control in other cities.
(2) The comparison of urban texture, POI, and distribution of COVID-19 shows that ① the overall functional layout of the city significantly impacts the COVID-19 distribution; ② there is a high risk of COVID-19 infection in places such as bus stops and schools; and ③ supermarkets and hotels have a high risk of secondary transmission and diffusion.
(3) The results of model training and model application show the following: ① When the urban texture and urban form elements are used as input data for learning, the stability and accuracy of the model learning results are good and high. ② Considering the cost saving of machine load and learning time, the prediction accuracy of the model after 200 iterations of learning and training can basically meet the target prediction requirements. ③ The prediction of the urban COVID-19 distribution through ML is more accurate than the distribution of the existing COVID-19 point map. Furthermore, the prediction results more clearly display the boundary scope of the COVID-19 distribution area, which is conducive to city managers for controlling the COVID-19 spread.

The spread of COVID-19 has caused the public to rethink the issue of public health governance. Method for predicting urban epidemic situations using machine learning and its potential for practical application:

(1) As an important reference for urban planning and design: Prediction of the possible risk areas of public health epidemics through CGAN could be used as a reference for design schemes. Architects and researchers can adjust the design of urban textures, such as building density, roads, and green spaces, based on the epidemic prediction results to reduce the intensity of epidemic risks.
(2) As a reference to urban public health governance and control: The urban texture and business data in different regions are different, and an epidemic prediction model can be established based on the actual data of different cities. The prediction results can be used to strengthen the city's epidemic prevention capabilities in a targeted manner, turn passive epidemic prevention into active epidemic prevention, and improve the efficiency of urban public health governance.
(3) As an issue of urban public health industry–university–research cooperation: Since the characteristics of each city differ, public health governance should “prescribe the right medicine.” Correspondingly, cooperation among local scientific research institutions, architectural design offices, medical and health prevention and control centers, etc., can be considered in the future to achieve the sustainable development of urban public health governance.
(4) For implementation in smaller areas, such as hospitals: In hospitals, a non-COVID-19 patient can be infected by other COVID-19 patients. If the footprints of the COVID-19 patients can be tracked and used for ML, the COVID-19 risk in the hospital can be predicted in real time. Areas can be designed to prevent other hospital patients from becoming infected and the workflow of medical staff can be adjusted.

## Data availability statement

The original contributions presented in the study are included in the article/Supplementary material, further inquiries can be directed to the corresponding author.

## Author contributions

LZ, YC, and JZ contributed to the topic selection, development of the framework, writing of the manuscript, figures, and the literature review. LZ and SJ contributed to the data analysis, drawings, and the tables. JS and YC contributed to the revision of data analysis and drawings. JZ contributed to the project administration and funding acquisition. All authors contributed to the article and approved the submitted version.

## Footnotes

^1^Macau is an inalienable part of China's territory, comprising the Macau Peninsula, Taipa, and Coloane, with a land area of 32.9 km^2^. The Macau Peninsula is connected to mainland China (Zhuhai City, Guangdong Province) in the north and to Taipa in the south by the Ponte Governador Nobre de Carvalho (Carvalho Bridge), the Ponte de Amizade (Friendship Bridge), and the Ponte de Sai Van (Sai Van Bridge). Taipa and Coloane are connected by a 2.2-km-long, 6-lane highway.

## References

[B1] Alsayed A. Sadir H. Kamil R. Sari H. (2020). Prediction of epidemic peak and infected cases for COVID-19 disease in Malaysia, 2020. Int. J. Environ. Res. Public Health 17, 4076. 10.3390/ijerph17114076 32521641 PMC7312594

[B2] Balak N. Inan D. Ganau M. Zoia C. Sönmez S. Kurt B.. (2021). A simple mathematical tool to forecast COVID-19 cumulative case numbers. Clin. Epidemiol. Glob. Health 12, 100853. 10.1016/j.cegh.2021.100853 34395949 PMC8352661

[B3] Bjørnstad O. N. Finkenstädt B. F. Grenfell B. T. (2002). Dynamics of measles epidemics: estimating scaling of transmission rates using a time series SIR model. Ecolog. Monogr. 72, 169–184. 10.1890/0012-9615(2002)072[0169:DOMEES]2.0.CO;2

[B4] Borji A. (2019). Pros and cons of gan evaluation measures. Comput. Vis. Image Understand. 179, 41–65. 10.1016/j.cviu.2018.10.009

[B5] Cao H. Feng J. Li Y. Kostakos V. (2018). Uniqueness in the city: Urban morphology and location privacy. Proc. ACM Interact. Mobile Wear. Ubiquit. Technol. 2, 1–20. 10.1145/3214265

[B6] Chen-Charpentier B. M. Stanescu D. (2010). Epidemic models with random coefficients. Math. Comput. Modell. 52, 1004–1010. 10.1016/j.mcm.2010.01.014

[B7] Chibbaro S Ganau M Todeschi J Proust F Cebula H. (2020). How SARS-CoV-2 is forcing us to reconsider and reorganize our daily neurosurgical practice. Neurochirurgie 66, 189–191. 10.1016/j.neuchi.2020.05.001 32405094 PMC7219419

[B8] Chrysos G. G. Kossaifi J. Zafeiriou S. (2018). Robust conditional generative adversarial networks. arXiv preprint arXiv:1805, 08657.

[B9] Cong W. Jie Y. Xu W. Min L. (2020). Analysis of early spatiotemporal spread of novel coronavirus pneumonia. Acta Physica Sinica 69, 243–252.

[B10] Dogan O. Tiwari S. Jabbar M. A. Guggari S. (2021). A systematic review on AI/ML approaches against COVID-19 outbreak. Complex Intell. Syst. 7, 2655–2678. 10.1007/s40747-021-00424-8 34777970 PMC8256231

[B11] Ganau M Netuka D Broekman M Zoia C Tsianaka E Schwake M. (2020). Neurosurgeons and the fight with COVID-19: a position statement from the EANS Individual Membership Committee. Acta Neurochir (Wien) 162, 1777–1782. 10.1007/s00701-020-04360-3 32472377 PMC7258601

[B12] Hao Y. Xu T. Hu H. Wang P. Bai Y. (2020). Prediction and analysis of corona virus disease 2019. PLoS ONE 15, e0239960. 10.1371/journal.pone.0239960 33017421 PMC7535054

[B13] Miranda J. G. V. Silva M. S. Bertolino J. G. Vasconcelos R. N. Cambui E. C. B. Araújo M. L. V.. (2021). Scaling effect in COVID-19 spreading: The role of heterogeneity in a hybrid ODE-network model with restrictions on the inter-cities flow. Physica D: Nonlinear Phenomena 415, p.132792. 10.1016/j.physd.2020.132792 33169041 PMC7641580

[B14] Mirza M. Osindero S. (2014). Conditional generative adversarial nets. arXiv preprint arXiv: 1411, 1784.

[B15] Ong J. Liu X. Rajarethinam J. Kok S. Y. Liang S. Tang C. S.. (2018). Mapping dengue risk in Singapore using Random Forest. PLoS Neglect. Trop. Dis. 12, e0006587. 10.1371/journal.pntd.0006587 29912940 PMC6023234

[B16] Pasaribu U. S. Mukhaiyar U. Huda N. M. Sari K. N. Indratno S. W. (2021). Modelling COVID-19 growth cases of provinces in java Island by modified spatial weight matrix GSTAR through railroad passenger's mobility. Heliyon 7, e06025. 10.1016/j.heliyon.2021.e06025 33659722 PMC7892810

[B17] Pei T. Wang X. Song C. Liu Y. Huang Q. Shu H.. (2021). Review on spatiotemporal analysis and modeling of COVID-19 pandemic. J. Geo-Inform. Sci 23, 188–210. 10.12082/dqxxkx.2021.20043433270029

[B18] Qian L. Yanni X. Jianhong W. Sanyi T. (2020). Construction of a COVID-19 epidemic time-lag model and analysis of confirmed case-driven tracing and isolation measures. Chin. J. Appl. Math. 43, 238–250.

[B19] Sanyi T. Yanni X. Zhixing P. Hongbing S. (2020). Predictive modeling of novel coronavirus pneumonia epidemic, data fusion and analysis of prevention and control strategies. Chin. J. Epidemiol. 41, 480–484.

[B20] Setti L. Passarini F. De Gennaro G. Barbieri P. Perrone M. G. Borelli M.. (2020). Airborne transmission route of COVID-19: Why 2 meters/6 feet of inter-personal distance could not be enough. Int. J. Environ. Res. Public Health 17, 2932. 10.3390/ijerph17082932 32340347 PMC7215485

[B21] Shen J. Liu C. Ren Y. Zheng H. (2020b). “Machine learning assisted urban filling,” in Proceedings of the 25th International Conference of the Association for Computer-Aided Architectural Design Research in Asia (CAADRIA), 2, 679–688. 10.52842/conf.caadria.2020.2.679

[B22] Shen Y. Yajie X. Xiaoying D. Xueye C. Jun Z. Han G.. (2020a). Spatial and temporal analysis of the spread of COVID-19 associated with geographic location. J. Wuhan Univ. Inf. Sci. Ed. 45, 798–807.

[B23] Shuo-zhong W. Xin-peng Z. (2007). Recent development of perceptual image hashing. J. Shanghai Univ. (English Edition), 11, 323–331. 10.1007/s11741-007-0401-2

[B24] Silva V. L. Heaney C. E. Li Y. Pain C. C. (2021). Data Assimilation Predictive GAN (DA-PredGAN): applied to determine the spread of COVID-19. arXiv preprint arXiv: 2105, 07729.36589258

[B25] Song Z. Lei S. Wei W. (2020). Analysis and evaluation of the development trend of the new crown epidemic in Jiangsu Province from the perspective of spatiotemporal big data. Modern Survey. Mapp. 43, 5–10.

[B26] Srinivas K. Bhandari A. K. Singh A. (2020). Low-contrast image enhancement using spatial contextual similarity histogram computation and color reconstruction. J. Franklin Inst. 357, 13941–13963. 10.1016/j.jfranklin.2020.10.013

[B27] Theis L. Oord A. V. D. Bethge M. (2015). A note on the evaluation of generative models. arXiv preprint arXiv: 1511, 01844.36135718

[B28] Wu J. T. Leung K. Leung G. M. (2020). Nowcasting and forecasting the potential domestic and international spread of the 2019-nCoV outbreak originating in Wuhan, China: a modelling study. Lancet 395, 689–697. 10.1016/S0140-6736(20)30260-9 32014114 PMC7159271

[B29] Yu Z. Wanli T. Zhongguang W. Zongwei C. Ji W. (2020). Propagation mechanism of COVID-19 along transportation routes based on improved SEIR model. Chin. J. Transport. Eng. 20, 150–158.

