# Supplementary material for: Predicting the distribution of COVID-19 through CGAN—Taking Macau as an example
Source: Front Big Data. 2023 Jan 25;6:1008292. doi: 10.3389/fdata.2023.1008292 (PMC9907848; doi:10.3389/fdata.2023.1008292)
Supplement: Supplementary file 1 [file Data_Sheet_1.docx]

Predicting the distribution of COVID-19 through CGAN—Taking Macau as an example

Liang Zheng ^1^†, Yile Chen †, Shan Jiang, Junxin Song, Jianyi Zheng ^*^,

†These authors contributed equally to this work and share first authorship

Faculty of Humanities and Arts, Macau University of Science and Technology, Macau, China

*** Correspondence:**Jianyi Zheng
jyzheng@must.edu.mo

# Appendix A

Machine learning environment configuration: the operating system is Windows 11 (X64), the Cuda version is 11.5, the deep learning framework is Pytorch, the graphics card is GeForce GTX 3070 (16G), and the processor is AMD Ryzen 9 5900HX (3.30 GHz).

# Appendix B(POI heat map of Macau Peninsula)


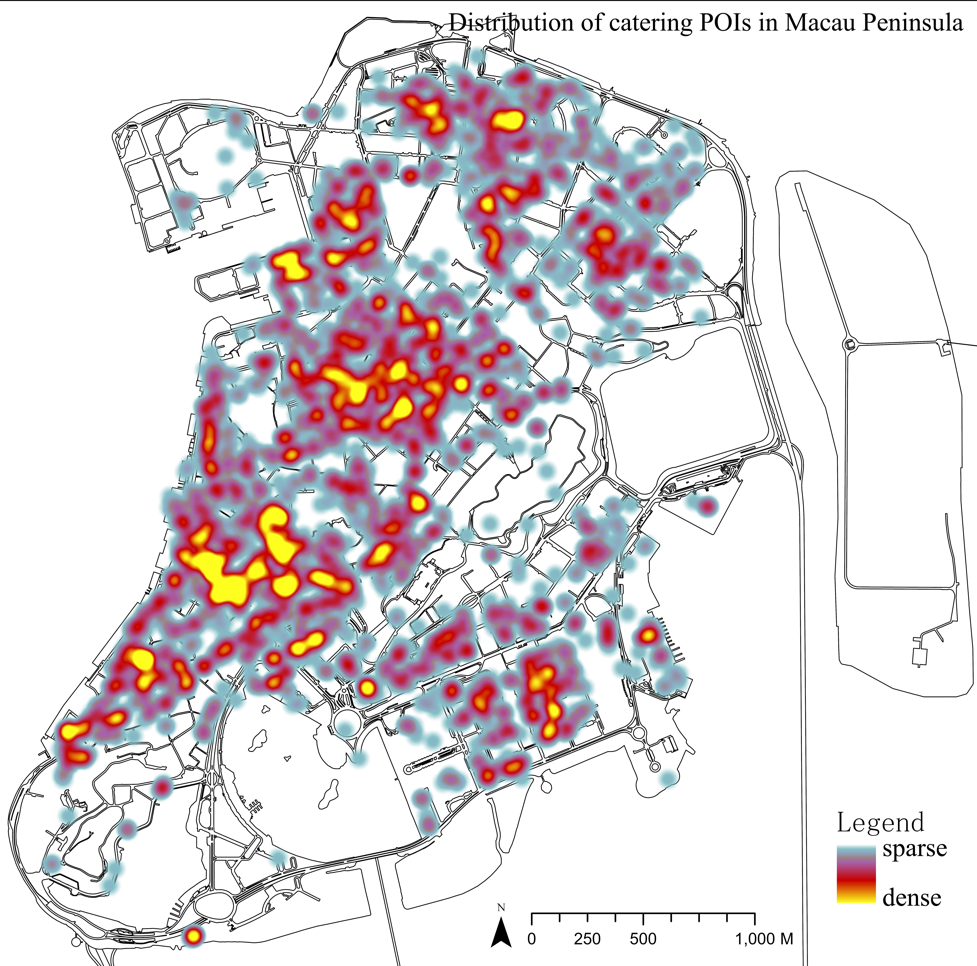


Distripution of catering POIs in Macau Peninsula


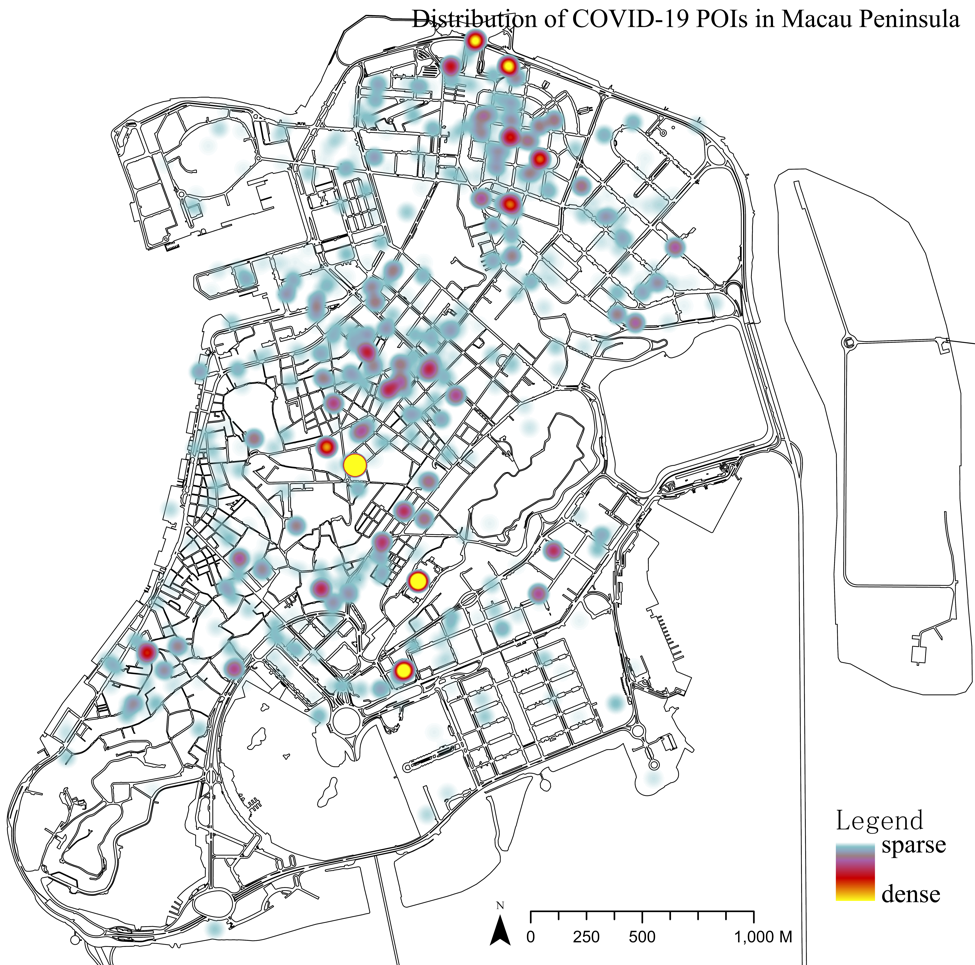


Distripution of COVID-19 POIs in Macau Peninsula


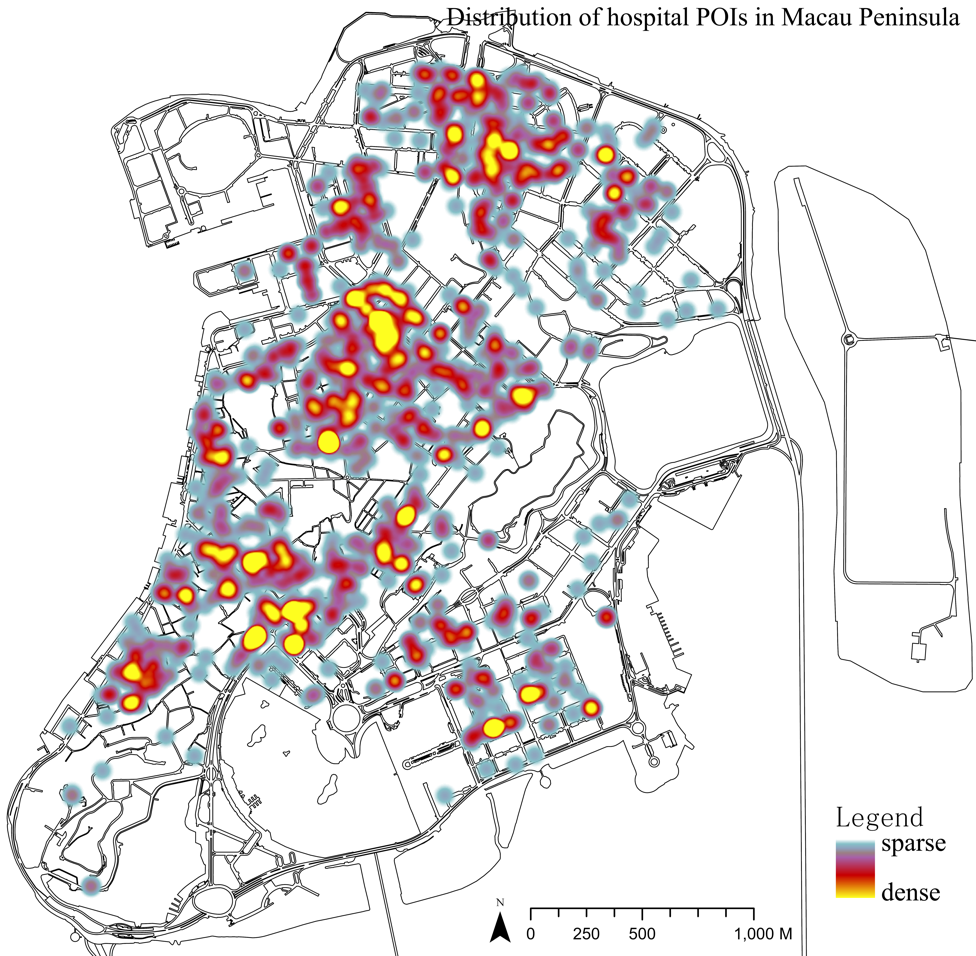


Distripution of hospital POIs in Macau Peninsula


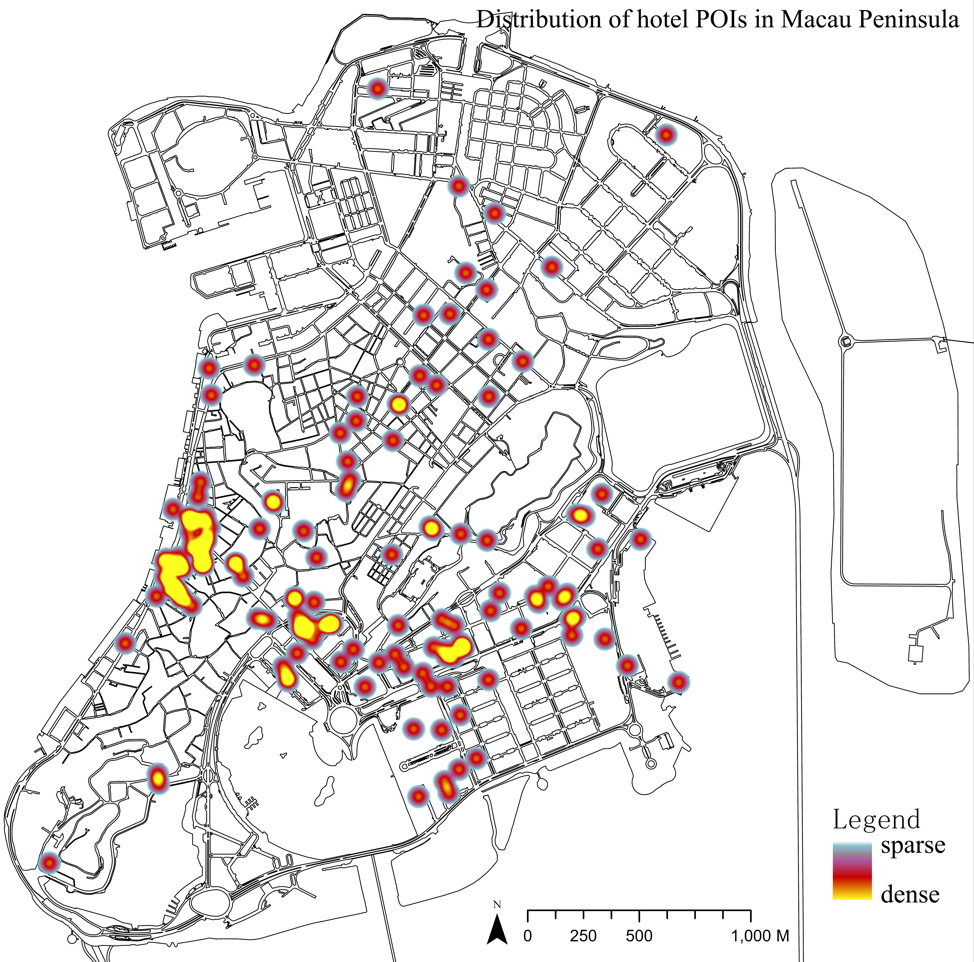


Distripution of hotel POIs in Macau Peninsula


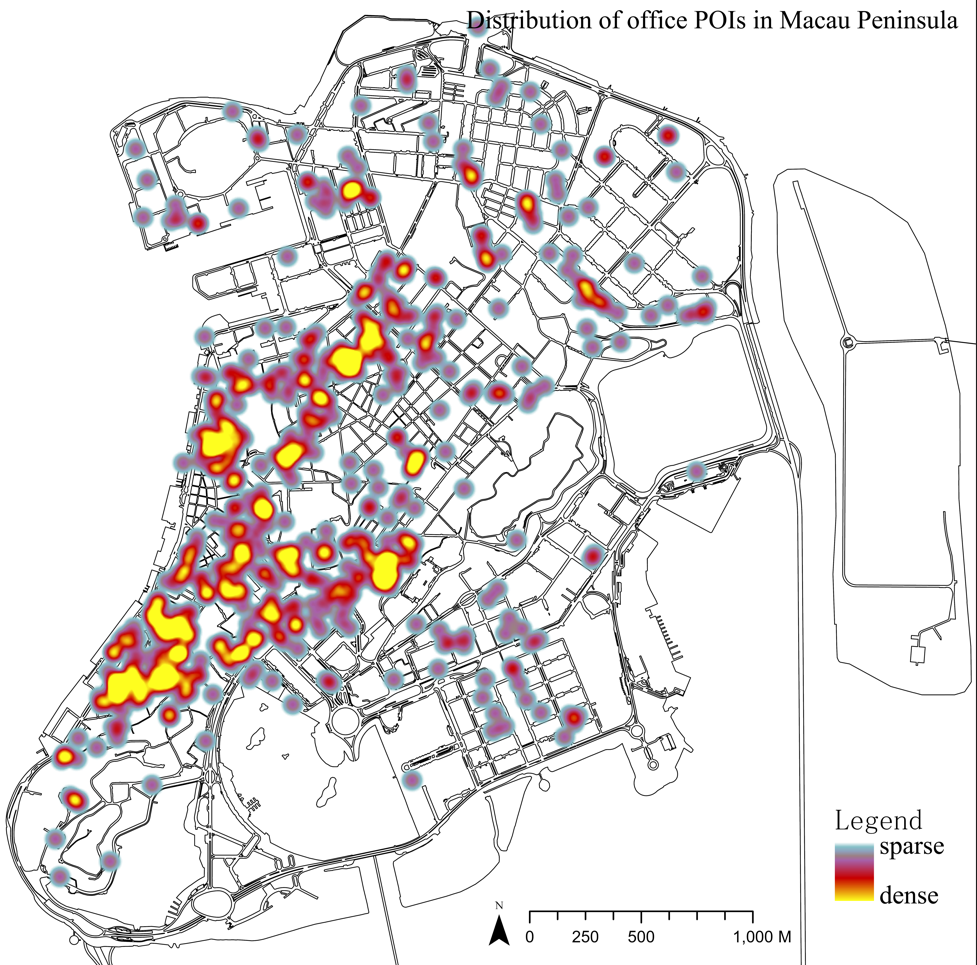


Distripution of office POIs in Macau Peninsula


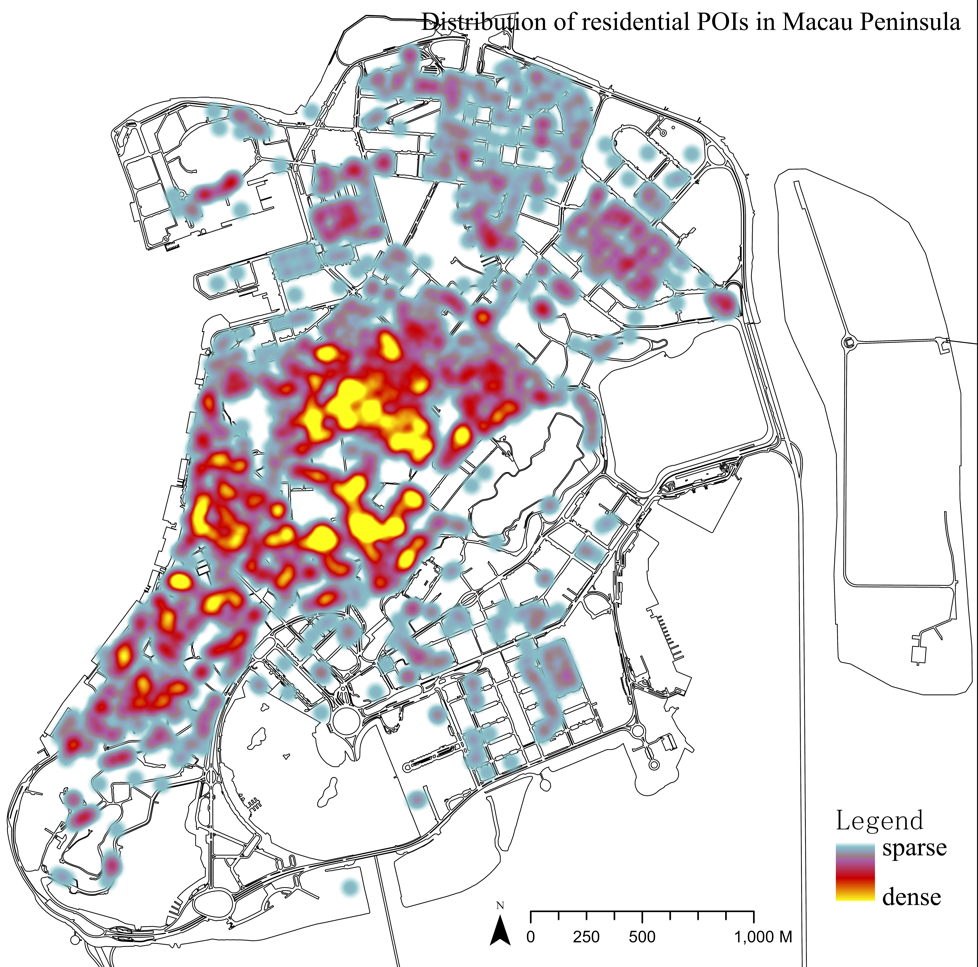


Distripution of residential POIs in Macau Peninsula


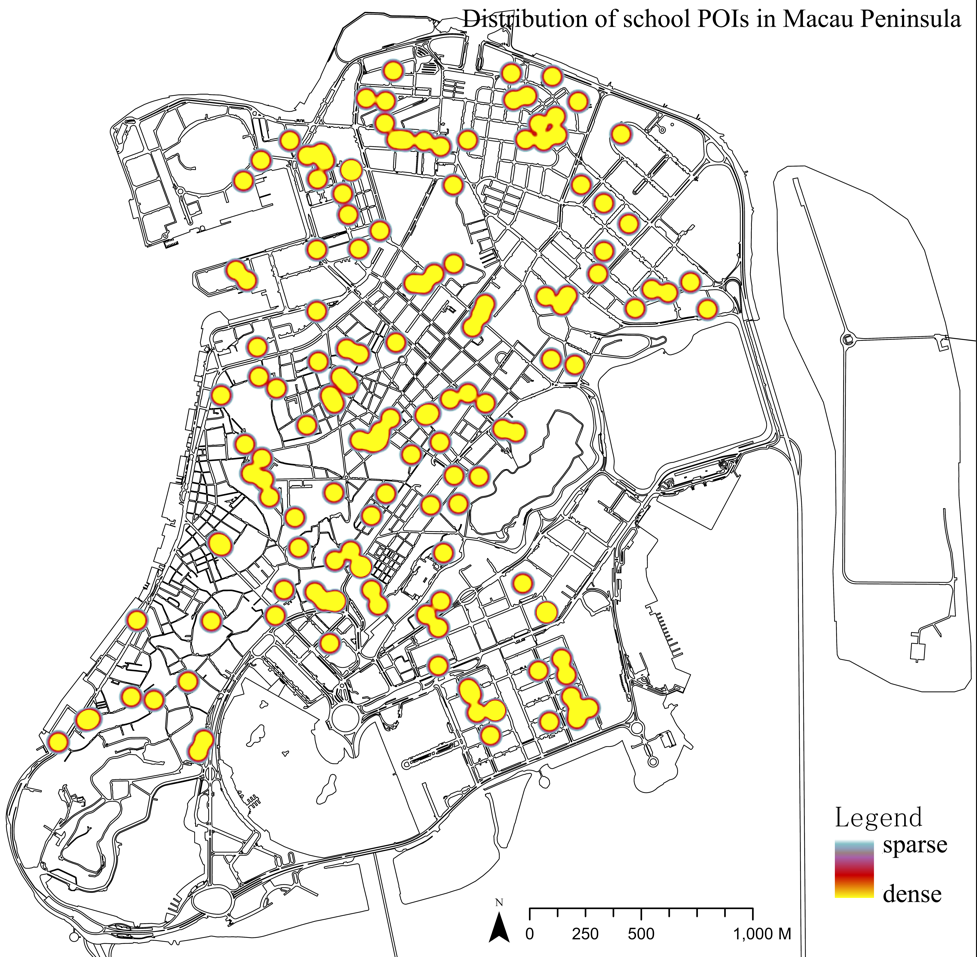


Distripution of school POIs in Macau Peninsula


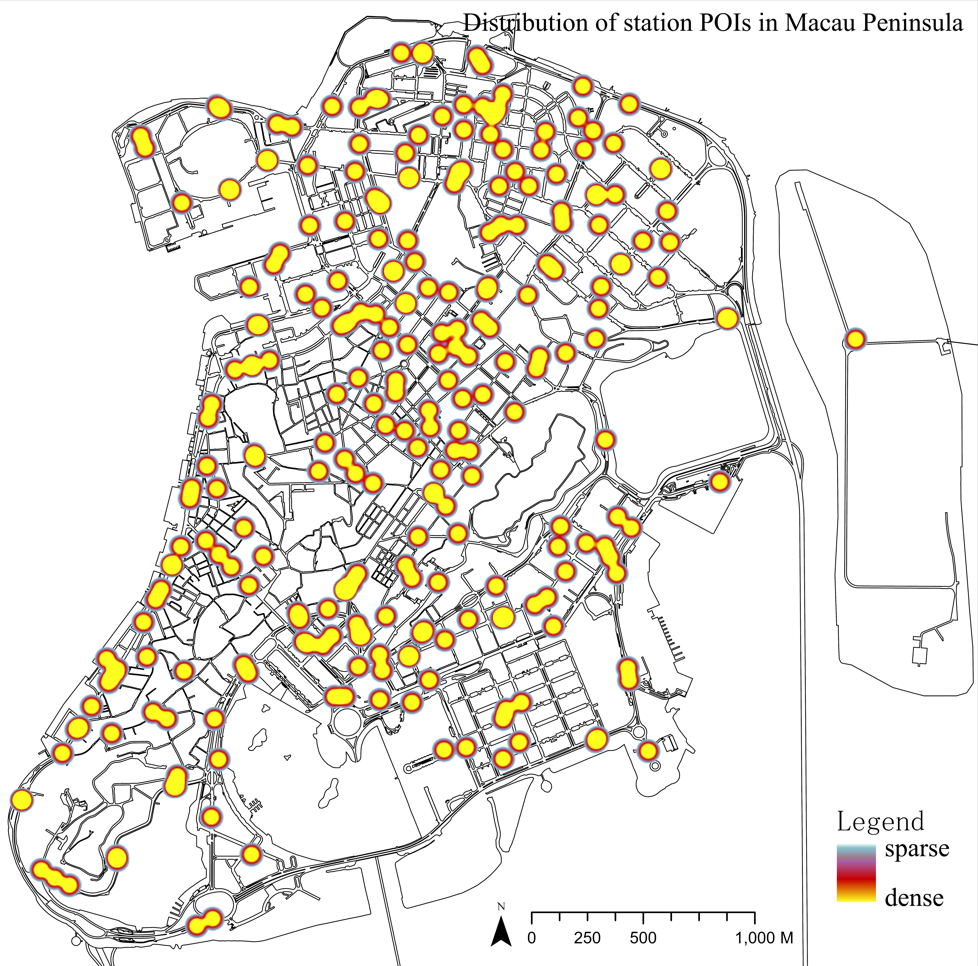


Distripution of station POIs in Macau Peninsula


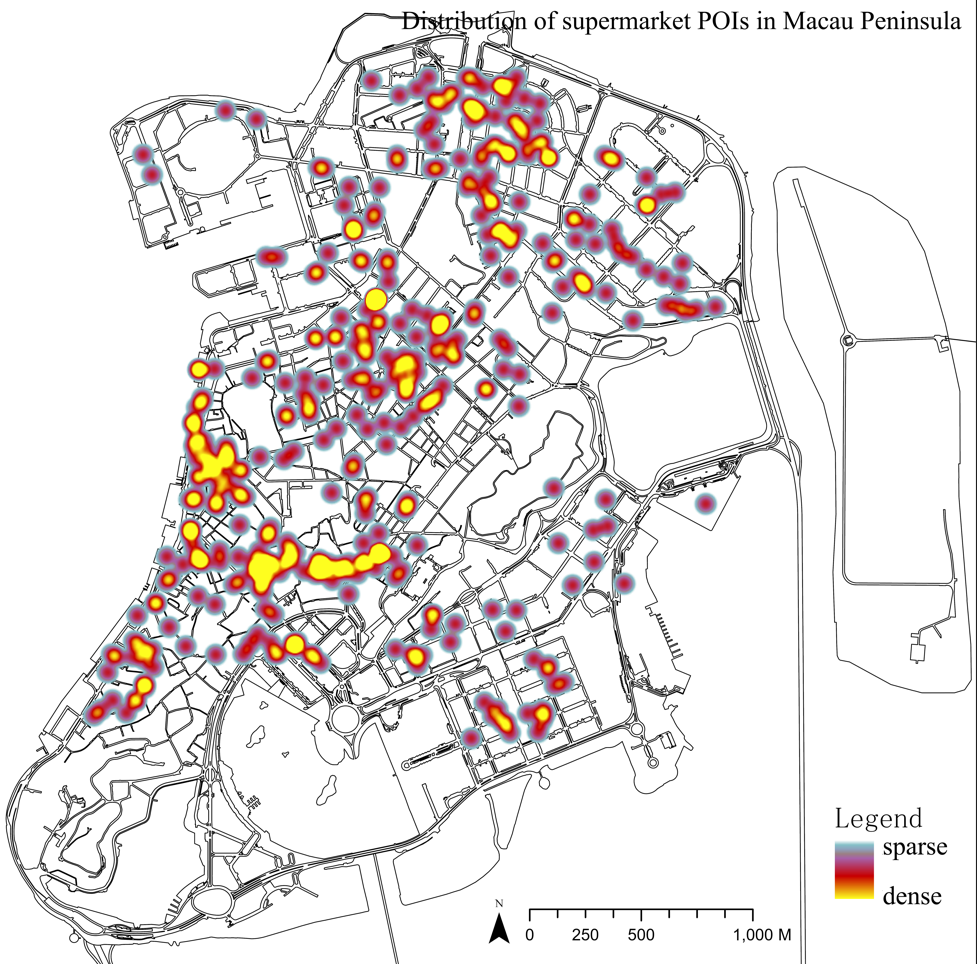


Distripution of supermarket POIs in Macau Peninsula
